# Contribution of walking to and from school on overall physical activity: a one-year follow up study

**DOI:** 10.1371/journal.pone.0318355

**Published:** 2025-03-12

**Authors:** Kensaku Sasayama

**Affiliations:** Faculty of Education, Mie University, Mie, Japan; Universiti Malaya, MALAYSIA

## Abstract

This study is the first in Japan to prospectively examine the relationship between walking to and from school and physical activity in primary school children. A total of 76 participants completed baseline and follow-up assessments, and their mean age was 9.6 ±  1.0 years at baseline and 10.6 ±  1.0 years at follow-up. The participants’ mode of school commute was measured by a questionnaire. Step counts, sedentary time, light physical activity (LPA) and moderate-to-vigorous physical activity (MVPA) was assessed using an accelerometer. Comparisons of physical activity variables at baseline and follow-up and tracking of physical activity were analyzed. Overall physical activity levels decreased at follow-up compared to baseline. However, the contribution of commuting school activities to overall physical activity significantly increased at follow-up compared to baseline, especially in step counts and MVPA. Walking to and from school contributed to the participants’ overall physical activity in MVPA were 39.6 ±  15.3% and 49.1 ±  13.8% for all participants at baseline and follow-up, respectively. Tracking correlation coefficients were high for the steps counts (r =  0.80–0.89) and MVPA (r =  0.71–0.75) in commuting school. In conclusions, walking to and from school significantly contributed to overall physical activity in primary school students. Physical activity during the school commute in short-term exhibited a low-to-strong association. These findings emphasize the importance of promoting physical activity interventions and implementing school policies that encourage walking to and from school. Future research will need to examine other populations and countries over a long-term period.

## Introduction

Physical activity has demonstrated positive associations with physical and mental health benefits in children [1,2]. Consequently, guidelines for children and adolescents aged 5–17 years emphasize the importance of engaging in at least 60 min of moderate-to-vigorous physical activity (MVPA) per day on average [3]. These recommendations are echoed by numerous countries as part of their physical activity guidelines [4]. However, globally, most adolescents aged 11–17 years fail to meet these guidelines [5].

In order to increase the rate of children walking to school, it is necessary to confirm longitudinal evidence of the contribution of walking to the physical activity per day. Several reviews have consistently reported that active modes of transport (e.g., walking and cycling) are associated with higher levels of physical activity [6–8]. However, walking to and from school rates are relatively low in many countries [9]. In contrast, Japan stands out according to the Global Matrix 4.0 Physical Activity Report Card [9] high rate of students walking to and from school. In the Global Matrix 4.0 of Report Card on physical activity from 57 countries [9], which ranks active transportation on a 13-point scale ranging from A to F, with Japan tied with Denmark for the highest rating of A − (80%–86% of children use active transport). In particular, the percentage of Japanese children walking to school is very high. A survey of all 1,095,282 fifth grade primary school students in Japan reported walking to school rates of 92.4% for boys and 93.5% for girls (Japan Sports Agency. 2018). This higher rate of walking to and from school is likely to result in a higher population-level MVPA. Indeed, Sasayama et al. [10] assessed physical activity using accelerometers in Japanese primary school children and reported that before- and after-school activities, including walking to and from school, accounted for 46.2% of step counts and 45.3% of MVPA. Furthermore, considering that MVPA peaks at age 5 or 6 and decreases with age [11,12], the contribution of walking to school may also increase with age. Martin’s review [8] showed that the contribution of school commuting is higher for high school students compared to primary school students, with a contribution of 23% and 36% for primary and high school students, respectively.

However, there are relatively few reports that have examined the relationship between active transport and physical activity longitudinally. Larouche’s review [7] on the relationship between active transport and physical activity highlighted a limited number of longitudinal studies. Out of the 49 articles reviewed, only 5 were prospective studies, and 2 were randomized controlled trials, 2 were quasi-experimental studies, and the majority (n =  40) was cross-sectional. Later, in 2023, Campos-Garzón et al. [13] reviewed the contribution of walking to school on physical activity, however only two of the 14 articles reviewed were longitudinal studies.

Therefore, it is crucial to investigate the prospective contribution of walking to and from school to daily physical activity. Confirming the prospective impact of walking to and from school is crucial as it would enhance the significance of walking to and from school for public health, particularly if it leads to higher population-level MVPA. Hence, this study examines longitudinally the relationship between walking to and from school and physical activity in primary school children.

## Methods

### Study design, setting, and participants

The participants in this study were used in a previous cross-sectional study by Sasayama et al. [10] that examined commuting to school and physical activity. This study analyzed the cross-sectional data of Sasayama et al. [10] with follow-up data in addition to the cross-sectional data of Sasayama et al. [10]. Therefore, the outcomes of commuting to school, physical activity, and anthropometry are the same as in the previous study by Sasayama et al. [10]. This prospective study was conducted in the city of Niimi, located in the northern area of Okayama Prefecture, Japan. Niimi has a population of approximately 30,000. All 17 primary schools in Niimi city were invited to participate in the study, and permission was obtained from the principal of one school in Niimi. The study was conducted from 1 April 2018 to 31 March 2021, with a one-year follow-up period to investigate modes of commuting to and from school and their association with physical activity. The survey was conducted over three years from 2018 to 2020, and longitudinal changes over one year (2018-2019, 2019-2020) were examined to avoid duplication of participants. In all years, the study was conducted in September. Initially, 104 consents were obtained at baseline, and at the one-year follow-up, 93 consents were obtained (dropout rate =  10.6%). Finally, the analysis included 76 participants (32 boys and 44 girls; mean age: 9.6 ±  1.0 years at baseline and 10.6 ±  1.0 years at follow-up) who participated at both baseline and follow-up, excluding those with missing data.

This study was approved by the Institutional Review Board of the Okayama University of Science (approval No. 29-3 and 31-3) and was conducted according to the principles of the Declaration of Helsinki. Written informed consent was obtained from all parents of the participating children before their involvement in the study.

### Mode of commuting to and from school

This study investigated all participants’ modes and duration of school commutes. The children reported their commute mode to and from school by completing a questionnaire. The modes of school commutes were assessed using two questions in Japanese. 1) “How do you usually travel to and from school?” with five response options including walk, bicycle, car, bus, or train. 2) “How long is your walk to and from school?” Children separately answered these questions for their commute to and from school and had the opportunity to select multiple commute mode if applicable. The participants answered the time required for all the modes of transport they used.

### Physical activity

Physical activity was assessed using an ActiGraph GT9X Link accelerometer (ActiGraph, LLC, Pensacola, FL, USA), which measured step counts, sedentary time, light physical activity (LPA), and MVPA. Participants wore the accelerometer on their waist for seven consecutive days, including weekends and weekdays, throughout the day, except during sleep, swimming, bathing, or contact sports. Although there is no consensus on the collection and processing of ActiGraph data [14], in this study, the accelerometer data were collected in 15-second epochs on at least three weekdays, and a valid wearing time of at least 600 min was ensured after excluding intervals of 30 min with zero counts. Sedentary time (<100 counts/min), LPA (101–2295 counts/min) and MVPA (≥2296 counts/min) were calculated using the cutoffs defined by Evenson et al. [15]. Only weekday data were used in this study.

In order to investigate physical activity during the school commuting period, the periods defined in previous studies before and after school were adopted in this study [16–18]. The before-school period was defined as the time between 6:00 am and 8:20 am (school start time). The end bell time (2:40–3:50 pm) varied based on grade and day of the week. Consequently, the after-school period was defined as the time from the end bell range until 5:00 pm. A valid wearing time of at least 60 min was required for both before- and after-school periods. Physical activity before and after school were calculated by averaging the mean physical activity during the before- and after-school period, respectively.

### Statistical analysis

The participants’ characteristics and physical activity variables at baseline and follow-up are presented as mean ±  standard deviation or number (%). As the normal distribution was not observed for several items of physical activity, the comparisons of physical activity variables at baseline and follow-up were analyzed using the Wilcoxon signed-rank test. The contribution of walking to and from school to the entire day was calculated by dividing the sum of the physical activity before and after school by the physical activity of the entire day and multiplying by 100. The tracking of physical activity was analyzed using Spearman’s correlation coefficient. Correlation coefficients ranging from 0.30 to 0.50, 0.50 to 0.70, and 0.70 to 0.90 indicated low, moderate, and high correlations, respectively [19]. All statistical analyses were performed using IBM SPSS Statistics software version 28 (IBM Japan, Ltd., Tokyo, Japan). Statistical significance was set at p <  0.05.

## Results

### Characteristics of the participants

Table 1 presents the characteristics of the participants, including age, mode of commuting to and from school, and time spent walking to and from school. The percentage of participants who reported commuting to and from school among all participants was 92.1% at the baseline and 96.1% at follow-up. The average time spent walking to and from school among all participants was 46.8 ±  21.4 min at baseline and 47.9 ±  21.4 min at follow-up.

**Table 1:** Characteristics of the participants.

	All (n = 76)						Boys (n = 32)						Girls (n = 44)					
	Baseline			Follow-up			Baseline			Follow-up			Baseline			Follow-up		
Age (years)	9.6	±	1.0	10.6	±	1.0	9.6	±	0.8	10.6	±	0.8	9.6	±	1.0	10.6	±	1.0
Mode of commuting to school																		
Walking	60		(78.9)	63		(82.9)	27		(84.4)	27		(84.4)	33		(75.0)	36		(81.8)
Bus or car	4		(5.3)	3		(3.9)	2		(6.3)	2		(6.3)	2		(4.5)	1		(2.3)
Walking and bus or car	12		(15.8)	10		(13.2)	3		(9.4)	3		(9.4)	9		(20.5)	7		(15.9)
Cycling	0		(0.0)	0		(0.0)	0		(0.0)	0		(0.0)	0		(0.0)	0		(0.0)
Train	0		(0.0)	0		(0.0)	0		(0.0)	0		(0.0)	0		(0.0)	0		(0.0)
Mode of commuting from school																		
Walking	59		(77.6)	63		(82.9)	27		(84.4)	27		(84.4)	32		(72.7)	36		(81.8)
Bus or car	6		(7.9)	3		(3.9)	2		(6.3)	2		(6.3)	4		(9.1)	1		(2.3)
Walking and bus or car	11		(14.5)	10		(13.2)	3		(9.4)	3		(9.4)	8		(18.2)	7		(15.9)
Cycling	0		(0.0)	0		(0.0)	0		(0.0)	0		(0.0)	0		(0.0)	0		(0.0)
Train	0		(0.0)	0		(0.0)	0		(0.0)	0		(0.0)	0		(0.0)	0		(0.0)
Time spent walking to school (min)	23.6	±	10.4	24.1	±	10.9	23.8	±	10.2	24.1	±	11.7	23.5	±	10.7	24.1	±	10.3
Time spent walking from school (min)	23.8	±	11.3	23.8	±	11.6	24.0	±	10.9	23.7	±	11.6	23.7	±	11.8	24.0	±	11.8
Time spent walking to and from school (min)	46.8	±	21.4	47.9	±	21.4	47.8	±	20.5	47.8	±	22.8	46.1	±	22.2	48.0	±	20.7

The values are presented as mean ±  standard deviation or number (percentage).

### Physical activity segments of the day and contribution of walking to and from school to the entire day

Table 2 presents the physical activity segments of the day and the contribution of walking to and from school for the entire day. Although step counts, sedentary time, LPA, and MVPA were significantly different between baseline and follow-up in before school and after school, only MVPA in the combined physical activity before and after school showed a significant increase at follow-up compared to baseline. For the entire day, only sedentary time showed a significant increase at follow-up compared to baseline. On the other hand, step counts and LPA in the entire day showed significant decreases at follow-up compared to baseline. The contribution of before and after school to the entire day showed a significant increase from baseline to follow-up in the step counts and LPA in all participants, boys and girls. These differences were statistically significant when the Wilcoxon signed-rank test was considered at the 95% confidence level.

**Table 2:** Physical activity segments of the day and contribution of walking to/from school to entire day.

	All (n = 76)								Boys (n = 32)								Girls (n = 44)							
	Baseline			Follow-up			z value	p value	Baseline			Follow-up			z value	p value	Baseline			Follow-up			z value	p value
Before school^1^																								
Step counts (steps)	2961.4	±	1110.1	2850.0	±	1063.7	-2.710	**0.007**	3018.2	±	1199.9	2921.8	±	1160.1	-1.683	0.092	2920.1	±	1052.3	2797.7	±	998.2	-2.171	**0.030**
Sedentary time (min)	45.7	±	16.6	47.4	±	17.4	-0.642	0.521	45.7	±	16.7	47.3	±	16.7	-0.280	0.779	45.7	±	16.8	47.4	±	18.1	-0.654	0.513
LPA (min)	34.2	±	9.6	31.3	±	9.7	-3.365	** < 0.001**	33.9	±	8.3	30.6	±	9.5	-2.375	**0.018**	34.5	±	10.6	31.8	±	9.9	-2.433	**0.015**
MVPA (min)	13.1	±	8.4	14.4	±	9.0	-1.631	0.103	14.4	±	8.4	16.8	±	9.7	-1.758	0.079	12.1	±	8.4	12.7	±	8.1	-0.502	0.616
After school^2^																								
Step counts (steps)	3083.6	±	1255.3	3473.0	±	1093.3	-3.402	** < 0.001**	3253.7	±	1448.2	3645.6	±	1159.7	-2.132	**0.033**	2959.9	±	1095.0	3347.5	±	1037.9	-2.672	**0.008**
Sedentary time (min)	41.7	±	10.1	39.0	±	9.1	-2.791	**0.005**	39.5	±	11.2	36.9	±	9.3	-1.832	0.067	43.3	±	9.0	40.5	±	8.7	-2.042	**0.041**
LPA (min)	36.2	±	10.1	36.4	±	7.9	-0.124	0.901	35.6	±	8.8	34.8	±	7.7	-0.486	0.627	36.7	±	11.1	37.5	±	7.9	-0.362	0.718
MVPA (min)	14.9	±	8.9	19.6	±	8.7	-4.551	** < 0.001**	17.0	±	9.9	22.7	±	9.1	-3.067	**0.002**	13.3	±	7.8	17.4	±	7.8	-3.221	**0.001**
Before and after school^1,2^																								
Step counts (steps)	6045.0	±	2226.5	6323.0	±	2014.7	-1.833	0.067	6271.9	±	2468.2	6567.4	±	2161.1	-1.234	0.217	5880.0	±	2046.5	6145.3	±	1906.8	-1.494	0.135
Sedentary time (min)	87.4	±	21.7	86.3	±	21.9	-0.564	0.573	85.2	±	22.8	84.2	±	21.2	-0.318	0.751	89.0	±	21.1	87.9	±	22.5	-0.420	0.674
LPA (min)	70.5	±	16.5	67.6	±	14.4	-7.574	0.072	69.5	±	13.4	65.4	±	13.3	-1.477	0.140	71.2	±	18.5	69.3	±	15.0	-1.027	0.304
MVPA (min)	27.9	±	16.2	34.0	±	16.3	-4.007	** < 0.001**	31.3	±	16.8	39.5	±	17.3	-3.011	**0.003**	25.4	±	15.4	30.1	±	14.5	-2.486	**0.013**
Entire day																								
Step counts (steps)	13076.0	±	3063.4	12658.6	±	2577.7	-2.061	**0.039**	14139.4	±	3385.9	13873.0	±	2697.8	-0.711	0.477	12302.6	±	2578.8	11775.4	±	2108.7	-2.077	**0.038**
Sedentary time (min)	517.0	±	70.5	547.2	±	88.0	-3.717	** < 0.001**	487.3	±	64.6	515.5	±	91.4	-1.832	0.067	538.7	±	67.3	570.2	±	78.7	-3.396	** < 0.001**
LPA (min)	257.3	±	39.8	243.3	±	32.6	-3.179	**0.001**	262.2	±	38.6	248.4	±	29.0	-1.702	0.089	253.6	±	40.8	239.6	±	34.8	-2.731	**0.006**
MVPA (min)	67.9	±	26.1	67.7	±	23.5	-0.280	0.780	78.1	±	27.3	80.4	±	23.7	-0.580	0.562	60.5	±	22.7	58.4	±	18.7	-1.091	0.275
Contribution of walking to/from school to entire day																								
Step counts (%)	45.5	±	11.3	49.4	±	10.4	-4.313	** < 0.001**	43.6	±	12.3	46.8	±	11.0	-2.412	**0.016**	46.8	±	10.4	51.2	±	9.7	-3.571	** < 0.001**
Sedentary time (%)	16.9	±	3.5	15.8	±	3.2	-2.547	**0.011**	17.4	±	3.6	16.4	±	3.3	-1.290	0.197	16.6	±	3.5	15.4	±	3.1	-2.532	**0.011**
LPA (%)	27.5	±	5.4	27.8	±	4.4	-1.025	0.305	26.7	±	5.0	26.2	±	3.6	-0.150	0.881	28.0	±	5.7	28.9	±	4.6	-1.342	0.180
MVPA (%)	39.6	±	15.3	49.1	±	13.8	-5.628	** < 0.001**	39.2	±	15.9	48.5	±	14.6	-3.571	** < 0.001**	39.8	±	15.1	49.5	±	13.3	-4.318	** < 0.001**

The values are presented as mean ±  standard deviation. ^1^Before school indicates from 6:00 am to school start time (8:20 am). ^2^After school indicates from school end bell time to 5:00 pm. LPA: Light physical activity. MVPA: Moderate-to-vigorous physical activity.

### Tracking of physical activity from baseline to follow-up by segments of the day

Table 3 shows the tracking of physical activity from baseline to follow-up by segment of the day. Among all the items of physical activity, consistent tracking patterns were observed for the majority of variables (r =  0.275–0.899, p <  0.05), with the exception of LPA. Among them, the tracking correlation coefficients were high for the steps counts (r =  0.799–0.889, p <  0.05) and MVPA (r =  0.713–0.745, p <  0.05) in before school and after school.

**Table 3:** Tracking of physical activity from baseline to follow-up by segments of the day.

	All (n = 76)		Boys (n = 32)		Girls (n = 44)	
	r	p values	r	p values	r	p values
Before school^1^						
Step counts	0.889	** < 0.001**	0.864	** < 0.001**	0.899	** < 0.001**
Sedentary time	0.515	** < 0.001**	0.446	**0.010**	0.556	** < 0.001**
LPA	0.671	** < 0.001**	0.585	**0.009**	0.722	** < 0.001**
MVPA	0.736	** < 0.001**	0.713	** < 0.001**	0.745	** < 0.001**
After school^2^						
Step counts	0.684	** < 0.001**	0.795	** < 0.001**	0.588	** < 0.001**
Sedentary time	0.550	** < 0.001**	0.478	** < 0.001**	0.516	** < 0.001**
LPA	0.275	**0.016**	0.277	0.124	0.237	0.121
MVPA	0.523	** < 0.001**	0.521	**0.002**	0.519	** < 0.001**
Before and after school^1,2^						
Step counts	0.821	** < 0.001**	0.820	** < 0.001**	0.799	** < 0.001**
Sedentary time	0.577	** < 0.001**	0.469	**0.007**	0.653	** < 0.001**
LPA	0.418	** < 0.001**	0.362	**0.042**	0.464	**0.002**
MVPA	0.730	** < 0.001**	0.720	**0.006**	0.715	** < 0.001**
Entire day						
Step counts	0.689	** < 0.001**	0.696	** < 0.001**	0.673	** < 0.001**
Sedentary time	0.738	** < 0.001**	0.574	**0.001**	0.708	** < 0.001**
LPA	0.513	** < 0.001**	0.309	0.085	0.592	** < 0.001**
MVPA	0.583	** < 0.001**	0.515	**0.003**	0.543	** < 0.001**

^1^Before school indicates from 6:00 am to school start time (8:20 am). ^2^After school indicates from school end bell time to 5:00 pm. LPA: Light physical activity. MVPA: Moderate-to-vigorous physical activity.

## Discussion

This one-year prospective study examined the contribution of walking to and from school in primary school children. The findings revealed that a high percentage of Japanese children engage in walking to and from school (Baseline: 94.7%, Follow-up: 96.1%). Moreover, walking to and from school contributed to the participants’ overall physical activity in step counts and MVPA were approximately 43.6%–51.2%, 39.2%–49.5%, respectively. Furthermore, significant correlations ranging from low to high (r =  0.459–0.728) were observed between physical activity before and after school from baseline to follow-up. These results underscore the significant contributions of walking to and from school to overall physical activity in children. Moreover, the findings highlight the importance of promoting physical activity among children who walk short distances to and from school and the need for supportive environments and school locations that facilitate walking to and from school.

The results showed that although some items significantly decreased the overall physical activity of the entire day (all participants and girls) from baseline to follow-up, physical activity during the commute to and from school did not significantly decrease in any of the outcomes or significantly increased in MVPA (Table 2). Furthermore, the contribution of walking to and from school to the entire day was higher in follow-up than in baseline for several physical activity outcomes. Cooper et al. [11] assessed physical activity with accelerometers in 27,637 participants (2.8–18.4 years) and reported an average decrease of 4.2% in total physical activity with age after 5 years of age. The results of present study may explain the decrease in total physical activity in previous studies [11], which occurred in domains other than walking to school. Indeed, while a decrease in the step counts of the entire day was also observed in present study, physical activity during the commute to and from school did not decrease over time after one year, and increased in MVPA. This phenomenon suggests that physical activity during the commute to and from school minimally decreases over time, and the relative importance of physical activity during the walk to and from school increases with age.

Dalene et al. [20] examined the association between longitudinal physical activity and walking to school and reported that active commuting was not significantly associated with changes in MVPA from age 9 to 15. Similarly, a longitudinal study by de Jesus et al. [21] examined the effect of active commuting modes on total physical activity and reported that walking to and from school had no significant effect on total physical activity, although bicycling was related. Ikeda et al. [22] also reported that the domains of active travel, organized sports, and physical education were not related to longitudinal changes in MVPA. In contrast to previous studies, the present study showed that walking to school contributes strongly to the total physical activity for the whole day, longitudinally. Some of these previous studies may have been influenced by factors such as assessing physical activity on the bicycle with an accelerometer, using a questionnaire to assess physical activity, and changes in the school stage during longitudinal follow-up. In contrast, Huang et al. [23] reported that a change from passive to active commuting was positively associated with a one-year change in MVPA among primary school students in Hong Kong. Furthermore, Werneck et al. [24] examined physical activity and commuting to school longitudinally in a large multinational study, which revealed that compared to active commuter children, passive commuter children reported higher declines in MVPA and were less likely to achieve recommended physical activity levels. This study clearly demonstrated the contribution of longitudinal walking to and from school to the entire day, although it was only a one-year prospective study. More long-term longitudinal studies on physical activity and active commuting to and from school are needed.

In this study, walking to and from school contributed to the participants’ overall physical activity in step counts and MVPA were approximately 43.6%–51.2%, 39.2%–49.5%, respectively. Martin et al. [8] reported that walking to and from school contributed to an average of 17 min/day (23% of total MVPA) based on data from 9 samples comprising a total of 3,422 participants. Similarly, Denstel et al. [25] conducted a study involving 6224 children across 12 countries and found that children who actively commuted to school accumulated an average of 6 min more MVPA per weekday compared to those who used a car. This study measured the time spent walking to and from school by the children as 46.8 ±  21.4 min, while the MVPA during the before- and after-school periods was measured as 27.9 ±  16.2 min. These findings indicate that the MVPA associated with walking to and from school in Japan is longer compared to international levels. One of the contributing factors to the high rate of walking to and from school in Japan is the deliberate placement of schools within a reachable distance of up to 4 km. Furthermore, it is common for children in Japan to walk to school alone [26], and the presence of a safe environment may play a role in promoting walking to school [27]. Previous study has reported that parents’ perception of safety on the way to school is related to the rate of decrease in active transport. Schools need to work with local governments to create a road environment that allows children to safely commute to school [28]. Policymakers should consider school locations and the safety of the neighborhood environment to promote physical activity among children.

Previous studies examining the tracking of daily physical activity have reported associations ranging from about r =  0.1 to 0.7. However, the degree of physical activity tracking varied depending on factors such as sex, age, years of follow-up, and physical activity intensity [29,30]. This study observed a significant correlation ranging from moderate to high (r =  0.513–0.738, p <  0.05) between baseline and follow-up measurements of step counts, sedentary time, LPA, and MVPA for the entire day. Furthermore, this study found tracking coefficients of step counts (r =  0.821), LPA (r =  0.418), and MVPA (r =  0.730) specifically during the before- and after-school periods. Notably, the tracking of step counts and MVPA during the before and after school was higher than that the tracking for the entire day among both boys and girls. The results highlight the significance of walking to and from school, particularly for girls, who tend to be less physically active than boys. Moreover, this study observed higher tracking coefficients for both boys and girls before school compared to after school, indicating that walking to school plays a significant role in the physical activity levels during the morning period.

This study has several limitations. First, this study’s data only represents one primary school in a specific region and small samples, limiting the generalizability of the findings. Although the selected schools in this study were similar to the average walking-to-school rate in Japan, the lack of multiple schools reduces the representativeness of the data. In particular, the rate of Japanese children walking to school is higher than in other countries. In the future, it will be necessary to examine the contribution of walking to the overall physical activity in a day in various countries with different rates of walking to school in a longitudinal study. Second, the evaluation of commuting time to school relied on subjective assessment. While questionnaires have been commonly used in previous studies [31] to gauge commuting mode and time, it is important to note that the reported commuting time may encompass activities other than just walking to and from school. In Japan, it is common for children to go to and from school using the same route, but there is a possibility that this is not the case. This subjective measurement introduces a potential source of bias and inaccuracies in the study’s findings. Furthermore, this study had a one-year follow-up period; therefore, long-term follow-up studies are necessary to assess the sustained impact of this behavior on the participants. In addition, by taking into account factors related to physical activity, such as recess, extracurricular activities and physical education, it may be possible to more clearly identify the impact of walking to school on physical activity. However, a notable strength of this study is that it focused exclusively on participants that solely relied on walking for commuting to and from school, excluding the influence of bicycling. Using accelerometers to assess physical activity during cycling may underestimate the actual level of physical activity [32]. By focusing solely on walking, this study was able to provide a more accurate understanding of the contribution of walking to and from school in participants with a high prevalence of this behavior.

## Conclusions

This study has provided evidence of the significant contribution of walking to and from school to the overall physical activity of primary school students in Japan. Moreover, this study observed a range of associations, varying from low to high, between physical activity during walking to and from school and overall physical activity in the short term. These findings highlight the importance of implementing interventions that promote physical activity among children who walk short distances to school. Furthermore, there is a need for supportive school environments and strategic school placements that encourage and facilitate walking to and from school.

## Supporting information

S1 Dataset Dataset of all parameters from 32 boys and 44 girls. (XLSX)
